# has-miR-134-5p inhibits the proliferation and migration of glioma cells by regulating the BDNF/ERK signaling pathway

**DOI:** 10.18632/aging.205720

**Published:** 2024-04-04

**Authors:** Zeshang Guo, Pingxv An, Xinyu Hong

**Affiliations:** 1 Department of Neurosurgery, The First Hospital of Jilin University, Changchun, Jilin 130021, China

**Keywords:** hsa-miR-134-5p, BDNF/ERK signaling pathway, MMP2/9, glioma

## Abstract

Our research investigated the effects of hsa-miR-134-5p on glioma progression, focusing on its interaction with the BDNF/ERK signaling pathway. U251 and U87 cell lines were analyzed post-transfection with hsa-miR-134-5p mimics and inhibitors, confirming the miRNA’s binding to BDNF using dual luciferase assays. Q-PCR was employed to measure expression changes, revealing that hsa-miR-134-5p markedly inhibited glioma cell proliferation, migration, and invasion, as evidenced by CCK8, monoclonal formation, and Transwell assays. Scratch tests and Western blotting demonstrated hsa-miR-134-5p’s modulation of the BDNF/ERK pathway and associated decrease in MMP2/9 protein levels. Flow cytometry suggested that hsa-miR-134-5p might also block the G0/S phase transition. *In vivo* studies using nude mice corroborated the tumor-suppressing effects of hsa-miR-134-5p, which were negated by elevated BDNF levels. Comparative protein analysis across groups confirmed the pathway’s significance in tumorigenesis. Our findings identify hsa-miR-134-5p as a key molecule impeding glioma cell growth by curtailing the BDNF/ERK pathway, with the reversal by BDNF upregulation pointing to the potential of therapeutically exploiting the hsa-miR-134-5p/BDNF axis in glioma care.

## INTRODUCTION

The transcriptional classification of gliomas reported by The Cancer Genome Atlas (TCGA) defines 4 subtypes, Classical (CL), Mesenchymal (MES), Neural (NE), and Proneural (PN) [1], advancing our knowledge of improving glioma diagnosis and treatment. However, brain gliomas remain a serious threat to patients, especially the most aggressive type of glioblastoma (GBM) [2]. Due to the blood-brain barrier, it is difficult to administer drugs and the cure rate is low. With the development and popularization of biotechnology, the research heat of miRNAs is increasing, which also brings new opportunities for the cure of GMB. Brain-derived neurotrophic factor (BDNF) is an important regulator of brain circuit development, synaptic and neuronal network plasticity, nerve regeneration, and nerve protection. Up-regulation and down-regulation of BDNF levels in human blood and tissues are associated with neurodegeneration, nervous system, and even cardiovascular diseases [3]. BDNF is a very important component of the brain growth factor neuro nutrient family, which can exert biological functions by binding to myosin receptor tyrosine kinase B (TrkB). BDNF/ERK signaling pathway is involved in various pathophysiological stimuli (such as oxidative stress, inflammation, etc.) to induce nervous system damage [4]. It has been determined that the expression of BCAN is related to glioma cell invasion, adhesion, and tumor growth, and is a marker of the malignant degree of glioma [5]. When extracellular signaling molecules bind to cell receptors, they convert extracellular stimuli into intracellular signals, a process also known as intercellular signaling. Cell proliferation and cell death are highly regulated processes involving signal transduction pathways. Tumor cells have abnormalities in the molecular regulation involved in these signaling pathways and, as a result, develop uncontrolled proliferation and defects in the mechanisms of apoptosis [6]. The initial study mainly found that hsa-miR-134-5p is a carcinogenic miRNA and its expression level is significantly increased in a variety of tumors [7–9]. This study explored the effect and mechanism of hsa-miR-134-5p on glioma based on the existing foundation.

## MATERIALS AND METHODS

Glioblastoma cell lines U251 and U87 were inoculated in DMEM medium containing fetal bovine serum (Dulbecco’s modified Eagle medium) and routinely cultured at 37°C under 5% CO_2_. Normal human astrocytes (NHAs) were purchased from ScienCell Research Laboratories, Inc. (San Diego, CA, USA) and cultured in the astrocyte medium (ScienCell Research Laboratories, Inc.). Both hsa-miR-134-5p mimic and hsa-miR-134-5p inhibitor, pcDNA3.1 plasmid vector BDNF-OE, and BDNF-NC were synthesized by Shanghai Sangon Biotech (Shanghai, China). Hsa-miR-134-5p primer design and synthesis (Shanghai Sangon Biotech Co., Ltd.). MEM medium (Gibco, USA: 42360032). Fetal bovine serum (Gibco: 10099-141). Dual antibody (Gibco: 15140-122). Trypsin solution (Gibco: 25200-056). Lipofectamine 3000 (Invitrogen, USA: L3000001). TRIzol (Invitrogen: 15596026). PrimeScript RT Master Mix (TaKaRa, Japan: RR036A). TB Green Premix Ex Taq (TaKaRa: RR420A). BCA protein concentration assay kit (Beyotime, China: P0012). CyclinD1 (Abcam, UK: ab16663). CyclinA2 (Abcam: ab181591). MMP2 (Abcam: ab92536). MMP9 (Abcam: ab76003). BDNF (Abcam: ab108319). p-TrkB (Abcam: ab229908). p-ERK1/2 (Abcam: ab184669). t-ERK1/2 (Abcam: ab17942). GAPDH (Abcam: ab8245). Goat anti-mouse secondary antibody (Abcam: ab97080) constant temperature and humidity cell incubator (Thermo Fisher Scientific, USA: Form371). Transwell Chamber (BD Company, USA). Velocity 18R Pro multi-functional desktop high-speed refrigerated centrifuge (Techcomp Laboratory Equipment (Shanghai) Co., Ltd., China). Azure C300 chemiluminescence imaging system (Shanghai Dowsontec Industrial Co., Ltd., China). Mini-Protean Tetra small vertical electrophoresis tank 165-8001 electrophoresis apparatus (Bio-Rad, USA).

### Cell culture and transfection

Transfection procedures in cells were carried out using Lipofectamine 3000, adhering strictly to the manufacturer’s protocol. Cells within the logarithmic phase were seeded onto 6-well plates until they achieved 75% confluency, after which the growth media were replaced with serum-free media. For the dilution of hsa-miR-134-5p mimic and inhibitor, as well as the Lipofectamine 3000 reagent, Opti-MEM medium was utilized. The control group received only the Lipofectamine 3000. The miRNA-liposome complexes were prepared at a 1:3 ratio and incubated at ambient temperature for 20 minutes. Post-incubation, cells were cultured at 37°C with 5% CO_2_ for 48 hours before Q-PCR analysis. Cells were then harvested at over 80% confluence in DMEM with fetal bovine serum for subsequent experiments.

### Real-time quantitative polymerase chain reaction (Q-PCR)

Primer sequences for the study were sourced from Sangon Biotech. Following cellular harvest, RNA was extracted from samples using TRIzol and reverse-transcribed into cDNA with PrimeScript RT Master Mix. RNA integrity was confirmed by NanoDrop ND-1000 spectrophotometry at 260 and 280 nm. Expression levels of hsa-miR-134-5p were quantified using Q-PCR with TB Green Premix Ex Taq and U6 as the internal reference, employing the 2^−ΔΔC^^T^ method for relative expression analysis.

**Table:** 

has-miR-134-5p-F	CGTGCTACAGTCCTGGTGAG
has-miR-134-5p-R	TACTCCATGACGCAGCAGTTGT
BDNF-F	CTTGGACAGAGCCAACGGAT
BDNF-R	GCAGCCTTCATGCAACCAAA
U6-F	CTCCGGCGCTTAGCACA
U6-R	AACTTACGAATCGCTTGCGT

### Dual luciferase

TargetScan software was used to identify BDNF as a putative downstream target of hsa-miR-134-5p. Luciferase assays in U251 cells involving either wild-type or mutated BDNF 3′-UTR sequences cloned into pmirGLO vectors were conducted to verify interaction effects. This was followed by triplet repeats of all experimental procedures and data acquisition from these iterative measurements.

### CCK8

Logarithmic phase U87 and U251 cells were dissociated, diluted, and quantified before being cultured at varying durations, 24 h to 72 h post-adhesion. Cell viability was subsequently assessed using a CCK-8 assay, with absorbance readings taken at 450 nm via a microplate reader, each measurement performed in triplicate.

### Clonal formation assay

Clonogenic assays involved treating logarithmic phase glioma cells with 0.25% trypsin, resuspending them in a complete medium, and performing gradient dilutions. Each cell group was seeded with 100 cells and maintained in a 37°C environment with CO_2_ saturation for two weeks until discernible clonal colonies formed. Subsequent processing included PBS washing, methanol fixation, and Giemsa staining, culminating in direct visual clone counting.

### Cell scratch assay

Wound healing assays began with 6-well plate glioma cell seeding, followed by scratch creation with a sterile pipette tip. The cells were cultured at regulated conditions, and healing progress was documented and analyzed at specified time points utilizing Image ProPlus software.

### Transwell assay

Enhanced Transwell migration assays were conducted with cells plated atop CeturegelTM Matrix LDEV-Free-coated membranes, allowing cells to migrate toward a lower-chamber FBS gradient. Afterward, migrated cells were quantified via crystal violet staining and inverted microscope imaging.

### Western blot

For immunoblotting, lysed cells were centrifuged to extract protein, quantified with a BCA kit, and separated using SDS-PAGE. The protein-laden membranes were incubated with primary antibodies specific to target proteins including CyclinD1, CyclinA2, MMP2, MMP9, BDNF, p-TrkB, ERK1/2, and the loading control GAPDH. After secondary antibody coupling, detection was facilitated through chemiluminescence.

### Flow cytometry

Cell cycle analysis involved ethanol fixation of transfected cells, PBS washing, and propidium iodide staining. Flow cytometry performed on a FACSalibur system, with Cell Quest software, was used to discern cell cycle stages.

### Tumor formation in BALB/c nude mice

The BALB/c Thymus nude mice (male, 4–6 weeks old) weighed 16–20 g. A xenograft tumor model of glioma cancer was established by subcutaneously inoculating 5 × 10^5^ U251 cells suspended in 100 μL phosphate-buffered saline into the wing of each nude mouse. After eight days, the mice were randomly divided into two groups with six mice in each group: hsa-miR-134-5p mimic or miR-NC (NC), hsa-miR-134-5p mimic plus BDNF-NC, and hsa-miR-134-5p mimic + BDNF-OE. These mimics were injected at a dose of 1 nmol per mouse into the implanted tumors every seven days for a total duration of four weeks. Tumor size was monitored by measuring length (L) and width (W) using calipers every four days and calculated using the formula: tumor volume = (L × W2)/2. On day 28, mice were euthanized via the cervical dislocation method, and tumors were quickly removed for protein analysis. Animal experiments were approved by the Animal Ethics Committee of the First Hospital of Jilin University.

### Statistical analysis

SPSS 25.0 and GraphPad were used for data analysis and processing. The measured data were expressed as mean ± standard deviation. The chi-square test was used for comparison between multiple groups, and the independent sample *t*-test was used for comparison between two groups. *p* < 0.05 was considered statistically significant.

### Data availability statement

The datasets generated during and/or analyzed during the current study are available from the corresponding author upon reasonable request.

## RESULTS

### Inhibition effect of hsa-miR-134-5p on the proliferation of glioma cells

**Figure 1:**
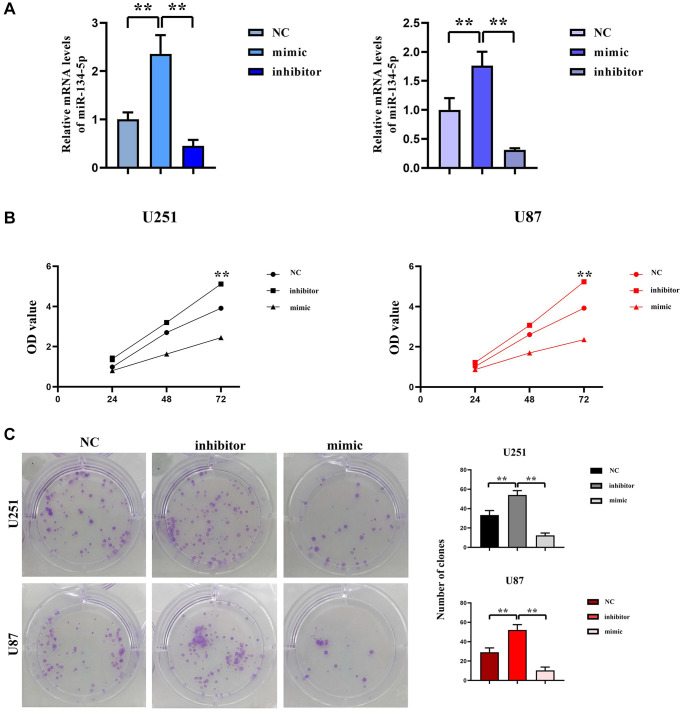
**Inhibition effect of hsa-miR-134-5p on the proliferation of glioma cells.** (**A**) The expression of hsa-miR-134-5p mimics and inhibitors was assessed by RT-PCR following transfection. (**B**) The cell proliferation capacity of U251 and U87 cells was assessed by CCK8 assay following transfection with hsa-miR-134-5p mimics and inhibitors. (**C**) The cell proliferation capacity of U251 and U87 cells was assessed by clonal formation following transfection with hsa-miR-134-5p mimics and inhibitors.

To elucidate the function of hsa-miR-134-5p in gliomas, both U251 and U87 cell lines were subjected to transfection with hsa-miR-134-5p mimics to augment the intracellular concentration of hsa-miR-134-5p, and conversely, its expression was mitigated through the application of hsa-miR-134-5p inhibitors. Post-transfection RT-qPCR analyses authenticated a marked upregulation of hsa-miR-134-5p in cells receiving miR-134-5p mimics, contrarily, a significant depression in expression was observed in cells administered with the hsa-miR-134-5p inhibitor, as presented in Figure 1A.

The proliferation effects attributable to the overexpression of hsa-miR-134-5p in glioma cells were examined utilizing the CCK8 assay. Cells infected with hsa-miR-134-5p evinced an amplified proliferative aptitude relative to the Mir-NC cohort, as depicted in Figure 1B. Parallel clone formation experiments indicated that overexpression of hsa-miR-134-5p curtailed the number of glioblastoma cell clones, whereas hsa-miR-134-5p downregulation led to an augmented clone count relative to Mir-NC-infected groups, delineated in Figure 1C. In summary, this evidence suggests the role of hsa-miR-134-5p in mitigating glioma cell proliferation.

### Inhibition effect of hsa-miR-134-5p on the migration and invasion ability of glioma cells

**Figure 2:**
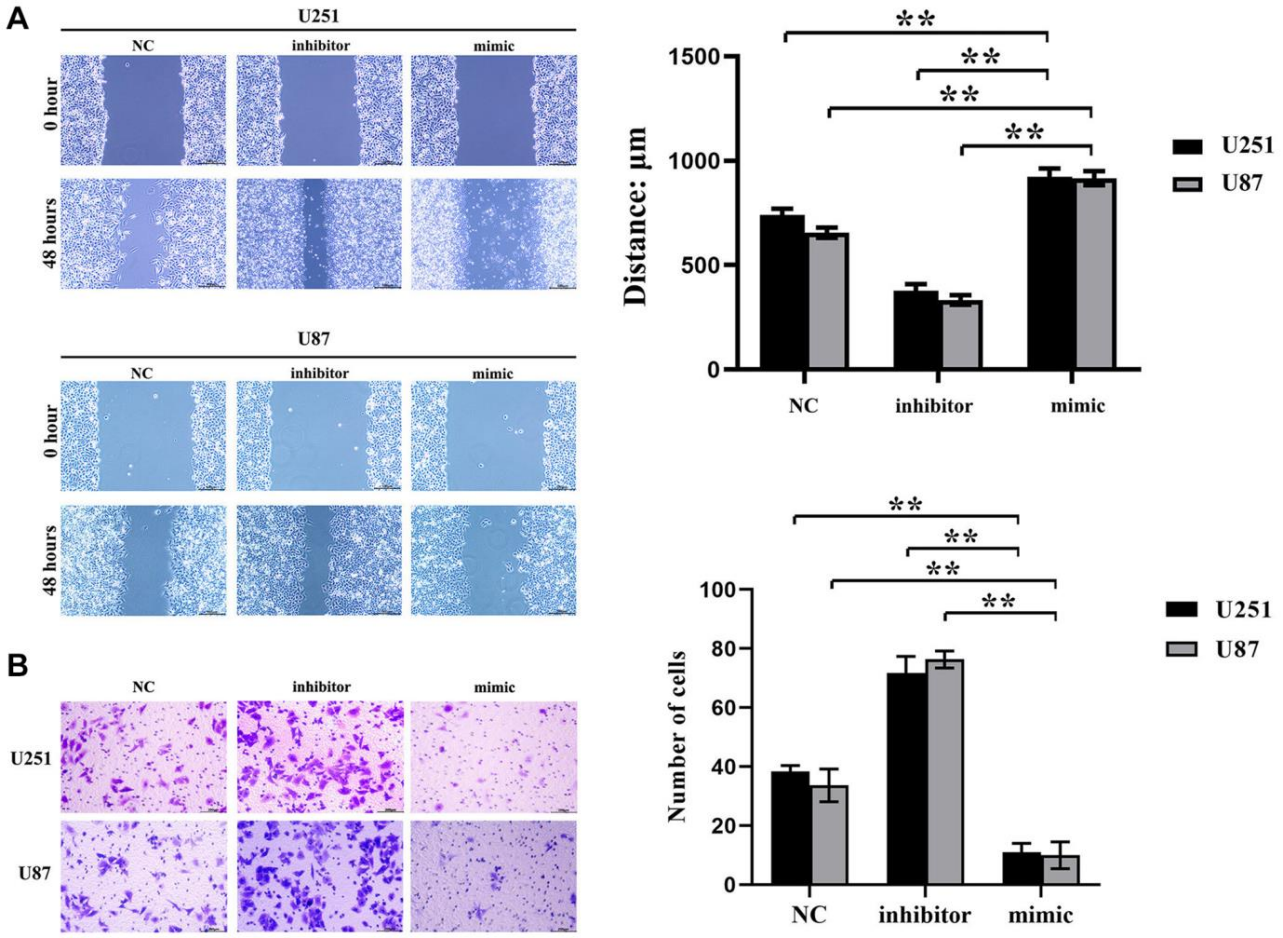
**Inhibition effect of hsa-miR-134-5p on the migration and invasion ability of glioma cells.** (**A**) The cell proliferation capacity of U251 and U87 cells was assessed by the scratch test following transfection with hsa-miR-134-5p mimics and inhibitors. (**B**) The cell proliferation capacity of U251 and U87 cells was assessed by Transwell following transfection with hsa-miR-134-5p mimics and inhibitors.

Cell scratch experiments exhibited that the induction of hsa-miR-134-5p mimics resulted in a considerable decrement of both migration distance and wound healing rate of U251 and U87 cells vis-à-vis the negative control (NC) group as showcased in Figure 2A. Moreover, hsa-miR-134-5p antagonism propelled an increase in both metrics. Accompanying Transwell invasion assays further substantiated a diminished cell invasion presence in hsa-miR-134-5p mimic-treated cells, as opposed to an elevated passage density in cells treated with the hsa-miR-134-5p inhibitor, amalgamating to denote the migration and invasion suppression properties of hsa-miR-134-5p in the evaluated glioma cells (Figure 2B).

### BDNF is a downstream target gene of hsa-miR-134-5p, which exerts its inhibitory effect on the cell cycle by suppressing the expression of proteins involved in cell cycle regulation and matrix proteases

**Figure 3:**
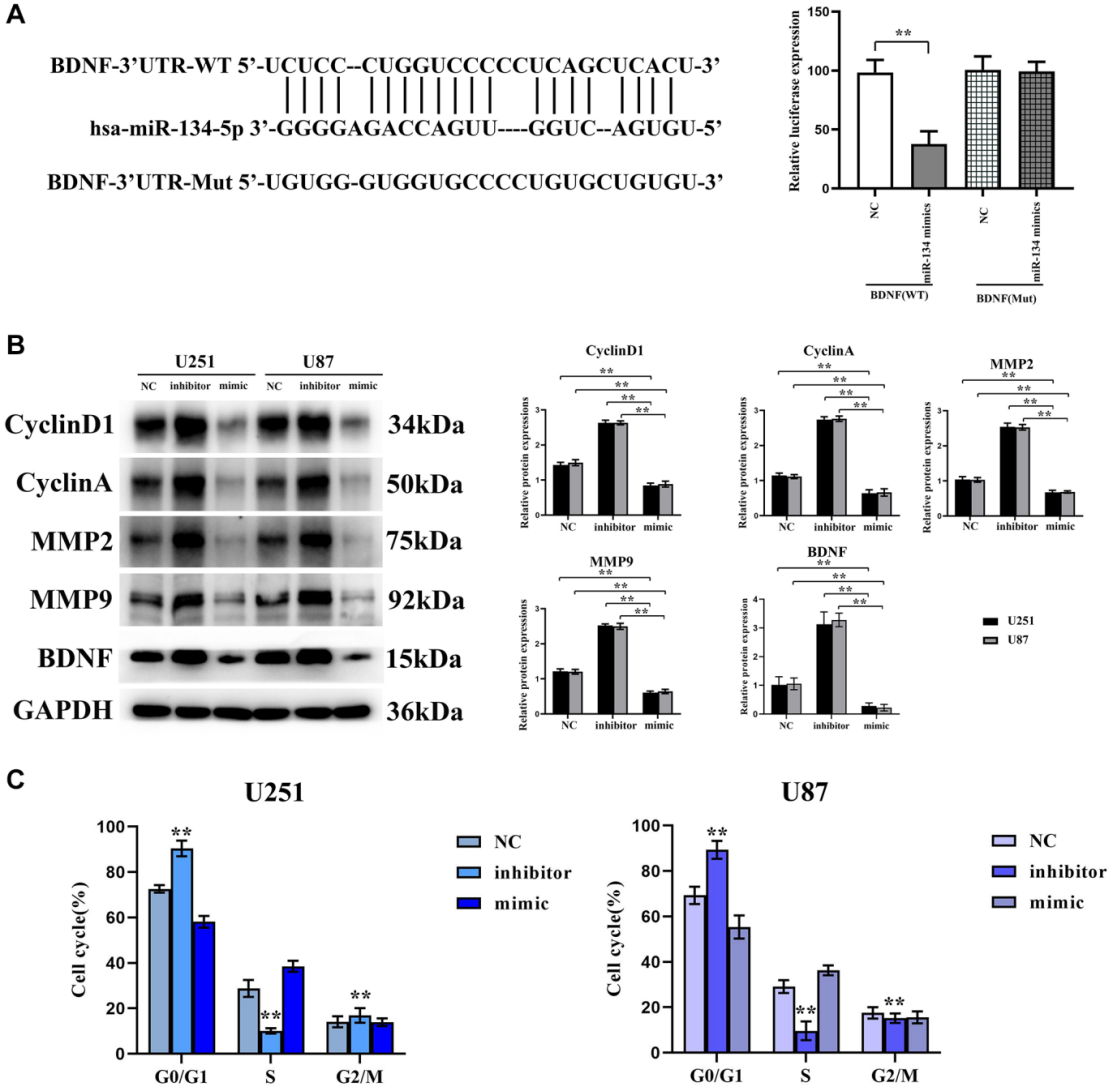
**BDNF is a downstream target gene of hsa-miR-134-5p, which exerts its inhibitory effect on the cell cycle by suppressing the expression of proteins involved in cell cycle regulation and matrix proteases.** (**A**) TargetScan to anticipate that BDNF would be the downstream gene of hsa-miR-134-5p. (**B**) Western blot shows the expression levels of cyclin D1, cyclin A2, and MMP2/9 were significantly reduced in U251 and U87 cells transfected with hsa-miR-134-5p mimics compared to those transfected with negative control. (**C**) Flow cytometry analysis revealed an increased proportion of G0/S phase cells in the hsa-miR-134-5p mimic group compared to cells transfected with NC. Conversely, a decreased proportion of G0/S phase cells was observed in the hsa-miR-134-5p inhibitor group.

BDNF was projected as a downstream target gene through the TargetScan algorithm, corroborated by luciferase reporter assays. These demonstrated a compelling suppression (~52%) of luciferase activity in U251 cells containing the wild-type BDNF 3′-UTR, compared to an insignificant effect within constructs housing mutant BDNF 3′-UTRs, as encapsulated in Figure 3A.

Concomitant Western blot assays manifested a substantive depletion of cyclin D1, cyclin A2, and MMP2/9 levels in U251 and U87 cells transfected with hsa-miR-134-5p mimics compared to the NC ensemble. Inversely, the expression of these proteins escalated within the hsa-miR-134-5p inhibitor group. Furthermore, an uptick in MMP2/9 was observed within the hsa-miR-134-5p mimic groups, suggesting an inhibition of glioma cell cycle progression influenced by hsa-miR-134-5p’s suppression of MMP2/MMP9 (Figure 3B). Flow cytometric analyses unveiled an inflated incidence of G0/S phase cells in the hsa-miR-134-5p mimic-treated cells, a stark contrast with the NC transfectants, augmenting the notion that hsa-miR-134-5p impedes cell division (Figure 3C).

### Effect of BDNF on ERK-related pathway protein expressions in glioma cells

**Figure 4:**
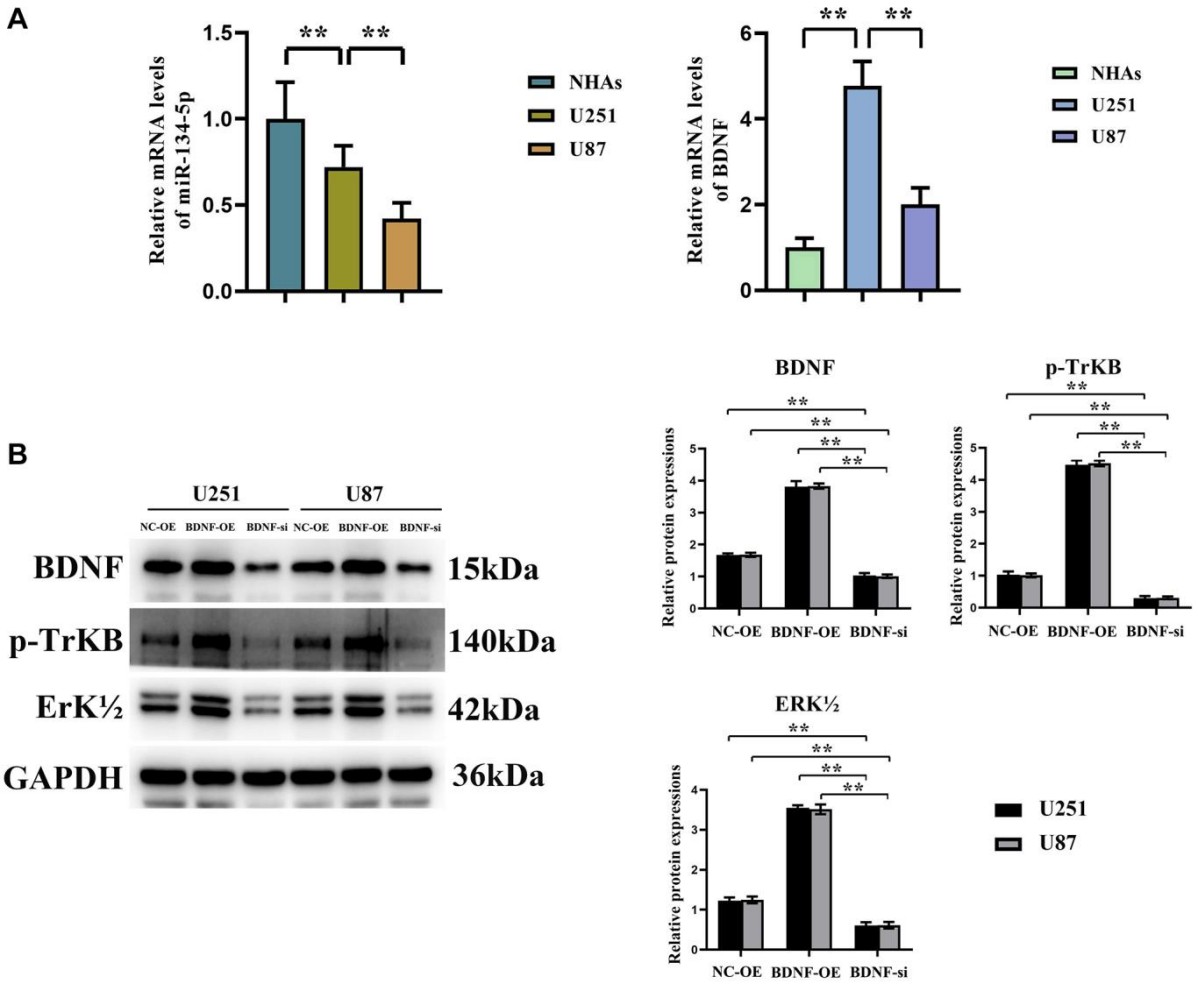
**Promoting Effect of BDNF on ERK-related pathway protein expressions in glioma cells.** (**A**) hsa-miR-134-5p is significantly downregulated and BDNF is significantly upregulated in detected by RT-PCR in glioma cells U251 and U87 cells, compared to Normal glioma cells. (**B**) Western blot shows expressions of related pathway proteins BDNF, p-TrKB, and ERK1/2 were significantly increased after oe-BNDF; expressions of related pathway proteins BDNF, p-TrKB, and ERK1/2 were significantly decreased after si-BNDF.

Quantitative RT-qPCR analysis disclosed the expression levels of hsa-miR-134-5p downregulation and BDNF upregulation mRNA across two glioma cell lines (U251, U87), a contrast to that observed in normal human astrocytes (NHAs) (Figure 4A). Delving further into BDNF’s mechanism, Western blot analyses revealed its overexpression to spur significant up-regulation in related ERK pathway protein expressions (BDNF, p-TrKB, and ERK1/2), whereas BDNF suppression provoked their down-regulation, with notable decrements in BDNF, p-TrKB, and ERK1/2 levels (Figure 4B). These data compellingly bolster the premise that BDNF modulates glioma progression via the ERK pathway and its associated substrates.

### hsa-miR-134-5p suppresses tumor growth in a nude mouse xenograft model by inhibiting BDNF

**Figure 5:**
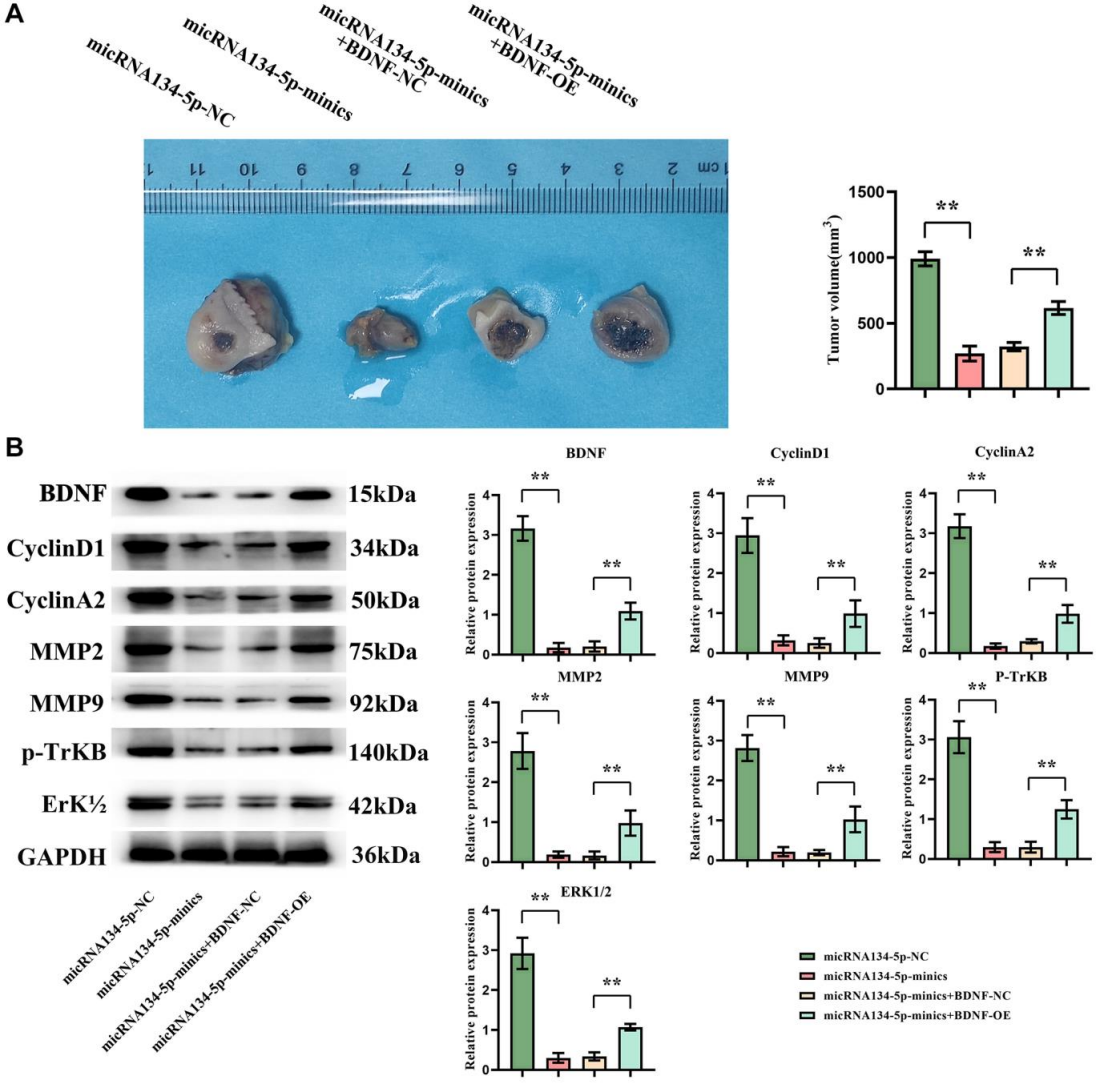
**hsa-miR-134-5p suppresses tumor growth in a nude mouse xenograft model by inhibiting BDNF.** (**A**) The nude mice exhibit tumor growth and a significant reduction in tumor volume in both the miR134-mimics group and miR134-mimics plus BDNF-NC group compared to the NC group; however, it should be noted that hsa-miR-134-5p mimics plus BDNF-NC treatment increased in tumor volume. (**B**) Western blot results revealed that the levels of BDNF, cyclin D1, cyclin A2, MMP2/9, p-TrKB, and ERK1/2 were significantly higher in both the miR134-mimics group and miR134-mimics plus BDNF-NC group compared to the NC group. However, in the miR134-mimics plus BDNF-OE group, there was an increase observed in the expression levels of BDNF, cyclin D1, cyclin A2, MMP2/9 as well as p-TrKB and ERK1/2.

Finally, to substantiate the *in vivo* tumoricidal effect of hsa-miR-134-5p, a BALB/c xenograft model was established employing U251 cells in nude mice, as delineated in the Methods. Observations distilled from this model tipped the scales towards a tangible diminution in tumor volume in subjects from both the miR134-mimics and miR134-mimics plus BDNF-NC groups vis-a-vis the NC group, the latter receiving an upswing in tumor volume post-treatment (Figure 5A). Western blot inspections correspondingly disclosed heightened expressions of BDNF, cyclin D1, cyclin A2, MMP2/9, p-TrKB, and ERK1/2 in the former groups, pinpointing a surge in the said proteins within the miR134-mimics plus BDNF-OE group (Figure 5B).

## DISCUSSION

The glioma is the most prevalent primary malignant brain tumor in adults, exhibiting significant morbidity and mortality rates. It can be classified into four grades based on its degree of malignancy [2]. Although surgical resection, radiotherapy, chemotherapy, and other comprehensive treatment modalities were implemented, the majority of patients exhibited an unfavorable prognosis. Consequently, the exploration of novel biomarkers for early detection has emerged as a pivotal endeavor to enhance the diagnosis and management of glioma [10–12].

The expression of hsa-miR-134-5p is downregulated in various tumor tissues and contributes to the malignant transformation of tumor cells. The decreased expression of hsa-miR-134-5p in human bladder cancer (BCa) is closely associated with advanced clinicopathological features, including higher tumor grade, histological grade, and T stage. It serves as a significant diagnostic and prognostic biomarker for BCa patients. Moreover, hsa-miR-134-5p exerts a pro-tumorigenic role in the progression of BCa cells [13]. The growth of CCA cells can be inhibited by hsa-miR-134-5p through the down-regulation of the Akt pathway, indicating that hsa-miR-134-5p has potential as a therapeutic target for cholangiocarcinoma (CCA) and could contribute to the development of effective treatment strategies [14]. The bioinformatics analysis predicted that hsa-miR-134-5p within bone marrow mesenchymal stem cells (MSCs) has the potential to target BDNF and bind to its 3′-UTR, thereby inhibiting the translation of BDNF protein [15]. The expression of hsa-miR-134-5p in pancreatic cancer tissues is significantly elevated compared to adjacent tissues. Overexpression of hsa-miR-134-5p in cancer cells exerts regulatory effects on BDNF and SEMA4C, thereby promoting the proliferation and invasion of SW1990 cells while inhibiting apoptosis [7]. The dual luciferase assay results from this experiment demonstrated the specific binding of hsa-miR-134-5p to BDNF. Furthermore, the overexpression of hsa-miR-134-5p was found to inhibit the proliferative activity and migratory capacity of glioma cells.

The secreted protein BDNF is expressed in both neuronal and non-neuronal cells. In neurons, BDNF immune reactivity can be observed in various regions of the central nervous system, as well as the peripheral and enteric nervous systems. In non-neuronal tissues, BDNF is synthesized in immune system cells such as T cells, B cells, and monocytes. Different mRNA variants can be transported to dendrites where local translation and release of BDNF may be selectively restricted to highly active dendrite stretching events, thereby facilitating synaptic plasticity through a BDNF-dependent mechanism [16]. BDNF belongs to the neurotrophic factor (NT) family, a closely related group of growth factors initially believed to be involved in the differentiation, proliferation, and survival of nerve cells in both the central and peripheral nervous systems. NTs are synthesized from a single protein precursor weighing approximately 27 kDa, known as preproto-NTS. This precursor consists of three domains: pre-maternal domain, prodomain, and mature domain. Sequential cleavage of these domains yields mature NT with a molecular weight of around 13 kDa. Immature NT (also referred to as parental NT) undergoes endoplasmic reticulum-mediated processing to generate proNT. Subsequently, proteolysis leads to the cleavage of pre-NT into mature NT. Intracellular division can occur through the action of proteins such as proconvertases or plasminases and matrix metalloproteinases (MMPs) [17]. BDNF effectively transmits signals via low-affinity receptors as well as high-affinity TrkB tyrosine kinase receptors [16]. MMP2 and MMP9, as members of the MMP family, play a crucial role in collagen and gel degradation, contributing to the proliferation and metastasis of various tumors [18–20]. The present study also confirmed the upregulation of MMP2 and MMP9 expression in cells exhibiting enhanced migratory and invasive properties, along with consistent ERK1/2 expression, thereby suggesting that hsa-miR-134-5p-mediated regulation of MMP2/9 occurs through the BDNF/TrkB/ERK1/2 pathway. Furthermore, studies have demonstrated that overexpression of hsa-miR-134-5p inhibits glioma cell migration by augmenting extracellular matrix protein hydrolysis, rendering tumor cells more prone to metastasis and further intensifying malignant transformation.

In conclusion, the upregulation of hsa-miR-134-5p exerts inhibitory effects on the proliferation, migration, and invasion of U251 and U87 cells through modulation of the BDNF/ERK1/2 signaling pathway, thereby playing a crucial role in suppressing glioma occurrence and progression. As a potential biomarker factor, hsa-miR-134-5p introduces novel therapeutic concepts and approaches for brain glioma treatment.

## References

[1] Verhaak RG, Hoadley KA, Purdom E, Wang V, Qi Y, Wilkerson MD, Miller CR, Ding L, Golub T, Mesirov JP, Alexe G, Lawrence M, O'Kelly M, et al, and Cancer Genome Atlas Research Network. Integrated genomic analysis identifies clinically relevant subtypes of glioblastoma characterized by abnormalities in PDGFRA, IDH1, EGFR, and NF1. Cancer Cell. 2010; 17:98–110. 10.1016/j.ccr.2009.12.020 20129251 PMC2818769

[2] Li Y, Ren Z, Peng Y, Li K, Wang X, Huang G, Qi S, Liu Y. Classification of glioma based on prognostic alternative splicing. BMC Med Genomics. 2019; 12:165. 10.1186/s12920-019-0603-7 31729991 PMC6858651

[3] Kojima M, Ishii C, Sano Y, Mizui T, Furuichi T. Journey of brain-derived neurotrophic factor: from intracellular trafficking to secretion. Cell Tissue Res. 2020; 382:125–34. 10.1007/s00441-020-03274-x 32897423

[4] Chen X, Xiao JW, Cao P, Zhang Y, Cai WJ, Song JY, Gao WM, Li B. Brain-derived neurotrophic factor protects against acrylamide-induced neuronal and synaptic injury via the TrkB-MAPK-Erk1/2 pathway. Neural Regen Res. 2021; 16:150–7. 10.4103/1673-5374.286976 32788470 PMC7818888

[5] Cook PJ, Thomas R, Kannan R, de Leon ES, Drilon A, Rosenblum MK, Scaltriti M, Benezra R, Ventura A. Somatic chromosomal engineering identifies BCAN-NTRK1 as a potent glioma driver and therapeutic target. Nat Commun. 2017; 8:15987. 10.1038/ncomms15987 28695888 PMC5508201

[6] Carrasco-García E, Saceda M, Martínez-Lacaci I. Role of receptor tyrosine kinases and their ligands in glioblastoma. Cells. 2014; 3:199–235. 10.3390/cells3020199 24709958 PMC4092852

[7] Qiu ZA, He GP. MicroRNA-134 functions as a tumor suppressor gene in gastric cancer. Am J Transl Res. 2016; 8:4320–8. 27830015 PMC5095324

[8] O'Brien K, Lowry MC, Corcoran C, Martinez VG, Daly M, Rani S, Gallagher WM, Radomski MW, MacLeod RA, O'Driscoll L. miR-134 in extracellular vesicles reduces triple-negative breast cancer aggression and increases drug sensitivity. Oncotarget. 2015; 6:32774–89. 10.18632/oncotarget.5192 26416415 PMC4741729

[9] Zhang X, Wang Y, Wang X, Zou B, Mei J, Peng X, Wu Z. Extracellular vesicles-encapsulated microRNA-10a-5p shed from cancer-associated fibroblast facilitates cervical squamous cell carcinoma cell angiogenesis and tumorigenicity via Hedgehog signaling pathway. Cancer Gene Ther. 2021; 28:529–42. 10.1038/s41417-020-00238-9 33235271

[10] Yang WB, Xing BZ, Liang H. Comprehensive analysis of temozolomide treatment for patients with glioma. Asian Pac J Cancer Prev. 2014; 15:8405–8. 10.7314/apjcp.2014.15.19.8405 25339037

[11] Badu S. Results of multiple resections in the treatment of anaplastic glioma. Bulletin of Neurology, Psychiatry, and Neurosurgery. 2022; 44–8.

[12] Schlömer S, Felsberg J, Pertz M, Hentschel B, Löffler M, Schackert G, Krex D, Juratli T, Tonn JC, Schnell O, Vatter H, Simon M, Westphal M, et al, and For The German Glioma Network. Mid-term treatment-related cognitive sequelae in glioma patients. J Neurooncol. 2022; 159:65–79. 10.1007/s11060-022-04044-1 35796933 PMC9325813

[13] Pan JY, Zhang F, Sun CC, Li SJ, Li G, Gong FY, Bo T, He J, Hua RX, Hu WD, Yuan ZP, Wang X, He QQ, Li DJ. miR-134: A Human Cancer Suppressor? Mol Ther Nucleic Acids. 2017; 6:140–9. 10.1016/j.omtn.2016.11.003 28325280 PMC5363400

[14] Huang M, Wang Y, Wang Z, Qin Q, Zhang H, Liu S, Cui J, Zhang Y, Jiang X, Xu L. miR-134-5p inhibits osteoclastogenesis through a novel miR-134-5p/Itgb1/MAPK pathway. J Biol Chem. 2022; 298:102116. 10.1016/j.jbc.2022.102116 35691339 PMC9257423

[15] Zampa F, Bicker S, Schratt G. Activity-Dependent Pre-miR-134 Dendritic Localization Is Required for Hippocampal Neuron Dendritogenesis. Front Mol Neurosci. 2018; 11:171. 10.3389/fnmol.2018.00171 29942249 PMC6004952

[16] Brigadski T, Leßmann V. The physiology of regulated BDNF release. Cell Tissue Res. 2020; 382:15–45. 10.1007/s00441-020-03253-2 32944867 PMC7529619

[17] Cao M, Niu Q, Xiang X, Yuan C, Iqbal T, Huang Y, Tian M, Zhao Z, Li C, Zhou X. Brain-Derived Neurotrophic Factor Regulates Ishikawa Cell Proliferation through the TrkB-ERK1/2 Signaling Pathway. Biomolecules. 2020; 10:1645. 10.3390/biom10121645 33302387 PMC7762527

[18] Zhang JF, Wang P, Yan YJ, Li Y, Guan MW, Yu JJ, Wang XD. IL-33 enhances glioma cell migration and invasion by upregulation of MMP2 and MMP9 via the ST2-NF-κB pathway. Oncol Rep. 2017; 38:2033–42. 10.3892/or.2017.5926 28849217 PMC5652951

[19] Jiang H, Li H. Prognostic values of tumoral MMP2 and MMP9 overexpression in breast cancer: a systematic review and meta-analysis. BMC Cancer. 2021; 21:149. 10.1186/s12885-021-07860-2 33568081 PMC7877076

[20] Chen S, Shen Z, Gao L, Yu S, Zhang P, Han Z, Kang M. TPM3 mediates epithelial-mesenchymal transition in esophageal cancer via MMP2/MMP9. Ann Transl Med. 2021; 9:1338. 10.21037/atm-21-4043 34532475 PMC8422148

